# Using Precision Medicine to Disentangle Genotype–Phenotype Relationships in Twins with Rett Syndrome: A Case Report

**DOI:** 10.3390/cimb46080497

**Published:** 2024-08-02

**Authors:** Jatinder Singh, Georgina Wilkins, Ella Goodman-Vincent, Samiya Chishti, Ruben Bonilla Guerrero, Federico Fiori, Shashidhar Ameenpur, Leighton McFadden, Zvi Zahavi, Paramala Santosh

**Affiliations:** 1 Department of Child and Adolescent Psychiatry, Institute of Psychiatry, Psychology and Neuroscience, King’s College London, London SE5 8AF, UK; jatinder.singh@kcl.ac.uk (J.S.); georgina.e.wilkins@kcl.ac.uk (G.W.); ella.goodman-vincent@kcl.ac.uk (E.G.-V.); samiya.chishti@slam.nhs.uk (S.C.); federico.fiori@kcl.ac.uk (F.F.); leighton.mcfadden@kcl.ac.uk (L.M.); 2 Centre for Interventional Paediatric Psychopharmacology and Rare Diseases (CIPPRD), South London and Maudsley NHS Foundation Trust, London SE5 8AZ, UK; 3 Centre for Interventional Paediatric Psychopharmacology (CIPP) Rett Centre, Institute of Psychiatry, Psychology and Neuroscience, King’s College London, London SE5 8AF, UK; 4 Genetica Consulting Services, Laguna Niguel, CA 92677, USA; ruben@geneticaconsulting.com; 5 Myogenes Limited, Borehamwood WD6 4PJ, UK; zahavi@myogenes.com

**Keywords:** Rett syndrome, genotyping, BDNF, precision medicine

## Abstract

Rett syndrome (RTT) is a paediatric neurodevelopmental disorder spanning four developmental stages. This multi-system disorder offers a unique window to explore genotype–phenotype relationships in a disease model. However, genetic prognosticators of RTT have limited clinical value due to the disorder’s heterogeneity on multiple levels. This case report used a precision medicine approach to better understand the clinical phenotype of RTT twins with an identical pathogenic *MECP2* mutation and discordant neurodevelopmental profiles. Targeted genotyping, objective physiological monitoring of heart rate variability (HRV) parameters, and clinical severity were assessed in a RTT twin pair (5 years 7 months old) with an identical pathogenic *MECP2* mutation. Longitudinal assessment of autonomic HRV parameters was conducted using the Empatica E4 wristband device, and clinical severity was assessed using the RTT-anchored Clinical Global Impression Scale (RTT-CGI) and the Multi-System Profile of Symptoms Scale (MPSS). Genotype data revealed impaired BDNF function for twin A when compared to twin B. Twin A also had poorer autonomic health than twin B, as indicated by lower autonomic metrics (autonomic inflexibility). Hospitalisation, RTT-CGI-S, and MPSS subscale scores were used as measures of clinical severity, and these were worse in twin A. Treatment using buspirone shifted twin A from an inflexible to a flexible autonomic profile. This was mirrored in the MPSS scores, which showed a reduction in autonomic and cardiac symptoms following buspirone treatment. Our findings showed that a combination of a co-occurring *rs6265 BDNF* polymorphism, and worse autonomic and clinical profiles led to a poorer prognosis for twin A compared to twin B. Buspirone was able to shift a rigid autonomic profile to a more flexible one for twin A and thereby prevent cardiac and autonomic symptoms from worsening. The clinical profile for twin A represents a departure from the disorder trajectory typically observed in RTT and underscores the importance of wider genotype profiling and longitudinal objective physiological monitoring alongside measures of clinical symptoms and severity when assessing genotype–phenotype relationships in RTT patients with identical pathogenic mutations. A precision medicine approach that assesses genetic and physiological risk factors can be extended to other neurodevelopmental disorders to monitor risk when genotype–phenotype relationships are not so obvious.

## 1. Introduction

Rett syndrome (RTT), a debilitating paediatric neurodevelopmental disorder, presents with a variety of symptoms spanning across four stages [1]. There is no cure for RTT. However, this multi-system disorder offers a unique window of opportunity to explore genotype–phenotype relationships in a disease model. About 90% of reported cases of RTT are due to mutations in the *methyl CpG-binding protein 2 gene* (*MECP2*), with frameshift and nonsense mutations being the most common [2]. While there is some evidence to suggest a genotype–phenotype correlation at the group level [3,4], RTT can be a clinical diagnosis based on clinical criteria that are independent of molecular profiles [5,6]. Even though 95% of patients with classic RTT have a pathogenic *MECP2* mutation, patients may also present with a pathogenic *MECP2* mutation without overt symptoms of RTT [7]. In the Findable, Accessible, Interoperable, and Reusable (FAIR) data set of the 10,968 *MECP2* variants examined, 863 were RTT-causing, while 209 were benign variants [8]. Reduced levels of MeCP2 can increase the risk of stress-induced mental health disorders in healthy women [9]. Additional evidence from a multi-site translational cohort study has also identified *MECP2* variants in females with precocious puberty without neurodevelopmental issues [10]. The genetic landscape of *MECP2* and its associative pathogenicity is complex, with more than 80 genes having been identified to exhibit RTT and RTT-like phenotypes [11]. This complexity is further compounded by X chromosome inactivation (XCI), which is suggested to be a driver for the variation in clinical severity in RTT [12]. Recent disease modelling of RTT has shown that 3% of the variation in the clinical severity seen in RTT is due to the interplay between paternal XCI and the *MECP2* variant [13]. When taken together, the evidence strongly suggests that genetic prognosticators in RTT and how they relate to the clinical phenotype should be interpreted carefully [14]. Early intervention and holistic management are needed for RTT patients to treat symptoms that are relatively straightforward before they become entrenched, chronic, and severe.

Precision medicine considers the individual’s genotype and environment to assist in managing different disorders. This approach may allow for a more accurate prognosis and treatment strategy optimisation focus. In the neurodevelopmental ecosystem, precision medicine has heralded a new era of therapeutics, and a cumulative approach relying on biopsychosocial–ecological, epigenetic, and cellular aspects provides a footing for delivering paediatric precision medicine [15]. This framework allows for the implementation of transdiagnostic assessment tools for the longitudinal examination of genetic and physiological risk factors that may extend beyond narrow diagnostic groups [16]. Using targeted genotyping and biometric physiological monitoring to examine the propensity of risk allows for a reframing of precision medicine in neurodevelopmental disorders. In the context of RTT, such an approach would be imperative due to the heterogeneity that exists at multiple levels from co-occurring disorders, polypharmacy, and the waxing and waning of symptoms across time as the disorder progresses. Objective physiological monitoring using wearable devices has allowed for examining sleep [17] and heart rate variability [18,19,20] in RTT patients. Other approaches have also used thermal imaging of skin temperature [21], polysomnographic (PSG) recordings and electrocardiograms [22,23], and variations between breathing and heart rate [24,25] during the sleep/wake cycle in RTT patients to study autonomic control. Further, clinical trials are underway, using other RTT biosensors for autonomic symptom tracking [26]. While studies on physiological measures can be helpful in developing biomarkers, this approach may be limited if a genetic strategy is not considered. Targeted genotyping combined with objective physiological monitoring may allow for a more robust multimodal approach to study neurodevelopmental disorders, especially when the interplay between genetic mutations and clinical outcomes are not so evident. A multimodal approach may also be better placed to stratify patients with neurodevelopmental disorders according to risk [27].

Twin studies can help further understand neurodevelopmental disorders’ aetiology [28]. Twin studies are rare in RTT. Previous evidence using videotaped observations from a RTT monozygotic twin has identified neurodevelopment abnormalities before regression [29]. Early behavioural interventions may also improve clinical outcomes in twin girls with RTT [30]. Another older study described two sisters with identical *MECP2* deletions but variable clinical phenotypes [31]. While some other twin studies of RTT have been documented [29], in the current case report, we assess the discordant clinical phenotype in twins with RTT that have the same pathogenic *MECP2* mutation. No study has used paediatric precision medicine that includes targeted genotyping combined with objective longitudinal physiological monitoring of heart rate variability (HRV) parameters to better understand the neurodevelopmental traits across different symptom domains in twins with RTT. The aim of the present case report is to assess whether a precision medicine approach can be used to characterise divergent neurodevelopmental trajectories in twins with RTT, with a view to disentangle the heterogeneity of clinical symptoms and severity.

## 2. Methods

### 2.1. Subject Demographics

Twin girls A and B (5 years 7 months old) were referred to the Centre for Interventional Paediatric Psychopharmacology (CIPP) Rett Centre for symptom management at 2 years 3 months of age. A documented pathogenic *MECP2* mutation for twins A and B was confirmed from a genetic report, as described previously [19]. Medical notes indicated that the twins were identical (monozygotic).

### 2.2. Buspirone Administration (Twin A)

Buspirone was started in July 2022 at a dose of 1.5 mg BD and titrated up to 2.5 mg BD for twin A. The dosing rationale was based on starting with the smallest dose possible and then titrating up to the optimum dose, in this case 2.5 mg BD. This dosing regimen was based on the effective dosing with minimum side effects (EDMS) strategy, as previously described [32].

### 2.3. Evaluation of Clinical Severity in Twin A and B

Hospitalisation (number of visits) was used as a proxy measure for clinical severity. This was coupled with the assessment of clinical severity using the RTT-anchored Clinical Global Impression Scale for Severity (RTT-CGI-S) [33] graded from normal (1), borderline impaired (2), mildly impaired (3), moderately impaired (4), markedly impaired (5), severely impaired (6) to extremely impaired (7). In addition, the frequency of symptoms was assessed using the caregiver-completed Multi-System Profile of Symptoms Scale (MPSS) [34]. The MPSS allows for a robust comparison of different symptom traits across various clinical domains in RTT. Twelve (12) validated subscales were used to capture information relating to autonomic problems, mental health, motor problems, respiratory issues, cardiac problems, neurological problems, gastrointestinal problems, orofacial, and sleep problems. Symptom frequency in the social behaviour, communication, and engagement domains were also assessed. The MPSS symptom frequency scoring was categorised as “not present” = 0, “rarely present” = 1, “sometimes present” = 2, “quite often present” = 3, “very often present” = 4, and “present all the time” = 5. Longitudinal RTT-CGI-S and MPSS symptom profiles for twins A and B were captured. Data were extracted and presented in Excel (Microsoft Excel, 2019).

### 2.4. Measurement of Heart Rate Variability Parameters

#### 2.4.1. Autonomic Heart Rate Variability Parameters

Heart rate variability (HRV) parameters were assessed to examine the impact of the autonomic nervous system (ANS) on clinical outcomes. For both twins A and B, the relative contribution from the sympathetic (SNS) and parasympathetic (PNS) components of the ANS was determined using the following time domain indices: pNN50 (percentage of successive R-R intervals that differ by more than 50 ms), SDNN (standard deviation of all NN intervals), and RMSSD (root mean square of successive differences) [35,36]. The average heart rate frequency (beats per minute, BPM) over time was also recorded.

#### 2.4.2. Measurement of Autonomic HRV Parameters

Longitudinal monitoring of HRV parameters was performed twice for each patient over a 12-month period (from January 2022 to December 2022). The Empatica E4 wristband was used to acquire the data for a continuous 24 h period. Empatica Manager Software (version 2.0.3) was used to upload the data from the E4 wristbands into the Empatica Connect cloud [37]. The sessions’ data were then downloaded from the Empatica Connect cloud into a secure encrypted hard drive.

Data management and analyses were performed using a custom-made script created on R ver. 4.3.2 language [38], in RStudio ver. 2023.09.0 + 463 [38]. Data for each session were extracted through Wearables package (Ver 0.8.1) [39] and processed using Tidyverse package [40]. The GGIR [41] formula was used to estimate asleep and awake periods based on accelerometer data that were modified to adapt to the thresholds for patients with RTT. Specifically, the angle of deviation of the accelerometer signal was progressively decreased to effectively identify a value which allows one to detect asleep/inactivity periods in this population which is frequently affected by movement disorders. The entire sessions captured for both the twins were classified using this algorithm for active/awake and asleep/inactive periods. This process generated one asleep and one awake sub-session for each time the patients wore the device. Further visual analyses of the signal were performed by an expert that identified possible artefacts in the accelerometer signal. Data affected by artefacts were excluded from further analyses (see Supplementary Information S1 for sub-sessions starting and ending times details).

Heart rate variability parameters were estimated using RHRV package (ver. 4.2.7) [42,43]. Inter-beat interval (IBI) data were used to estimate the HRV indexes in each of these data. The sub-sessions’ IBI was cleaned by applying the FilterNIHR function, which is the default NIHR filter of RHRV. Further analyses were performed by considering a five-hour portion of the data for each awake sub-session recorded using the E4 wristband. This procedure generated 4 sub-sessions (each of them of five hours), which were subsequently divided into 15 min consecutive sections of data. There were 20 consecutive 15 min sections of IBI data for each patient for each time they wore the E4 wristband. The HRV index values, which were more than two standard deviations from average values, were identified as outliers and excluded from further investigation. This allowed for the monitoring of the mean HR, SDNN, pNN50, and RMSSD over the 5 h time window.

### 2.5. Targeted Genotyping

#### 2.5.1. Sample Collection

Following parental consent, buccal swabs from twins A and B were obtained in December 2022 for genotype testing at the Centre for Interventional Paediatric Psychopharmacology (CIPP) Rett Centre. Myogenes (Myogenes, UK) sent the samples for analysis to Innovative Gx Health, which performed targeted genotype testing using next-generation sequencing.

#### 2.5.2. Targeted Genotype Reporting

Targeted genotyping was performed for *MECP2* and *BDNF*.

#### 2.5.3. *MECP2*

This genotyping for twins A and B was performed by an NHS Foundation Trust Genomic Laboratory using trio exome sequence analysis of the coding region and conserved splice sites of 23,244 genes by next-generation sequencing (Twist Core Human Exome/Illumina NextSeg), with a specificity greater than 99%. Confirmatory testing was performed by targeted junction fragment Sanger sequencing of *MECP2* and dosage analysis of exon 4 of *MECP2* (NM_004992.3) by Droplet Digital PCR (EvaGreen), using the Bio-Rad QX200 Droplet Digital PCR system. Variants were classified based on consensus recommendations from the American College of Medical Genetics and Genomics and the Association for Molecular Pathology [44].

#### 2.5.4. *BDNF*

The genotype reports for twins A and B had information and interactions for 67 genes and 294 medications, as described by expert working groups and regulatory organisations: Clinical Pharmacogenetics Implementation Consortium (CPIC), European Medicines Agency (EMA), Food and Drug Administration (FDA), Association for Molecular Pathology (AMP), College of American Pathologists (CAP), American College of Medical Genetics (ACMG), and Pharmacogenomics Knowledgebase (PharmGKB). This extended gene panel allows for targeting genotyping to explore the impact of other genes in RTT. For this case report, targeted genotyping was performed for brain-derived neurotrophic factor (*BDNF;* OMIM 113505), and the information on *BDNF* genotypes was extracted.

### 2.6. Statistical Analyses

The longitudinal monitoring of HRV parameters obtained in January and December 2022 was analysed with the non-parametric Wilcoxon signed-rank tests. We tested for possible differences between the two time points for each twin and the relative differences between twins at each time point. We performed the analyses for all the HRV parameters considered in this case report. To assess if the MPSS sub-scales scores for the twins differ from the overall RTT population, we used the reference data part from our previous study, in which we validated the MPSS [34] and performed a series of tests based on the Bayesian inferential method of the frequentist significance test [45]. The test used is based on Bayesian Monte Carlo methods that show a series of indexes which can be interpreted as a measure of similarity between the observed score and the reference population’s score. The results were interpreted as indicative of the symptom sub-groups’ evolution over time.

### 2.7. Informed Consent and Ethics

This case report forms part of the Tailored Rett Intervention and Assessment Longitudinal (TRIAL) Data Warehouse study. Study participants consented to the collection and use of the data in research and publications, and the study was approved by the London-Bromley Research Ethics Committee (REC reference: 15/LO/1772) on 19 October 2022 (substantial amendment number 7). Twins A and B underwent targeted genotype testing and longitudinal physiological monitoring as part of their routine clinical care.

## 3. Results

### 3.1. Longitudinal Monitoring of Clinical Severity and Symptom Frequency

**Figure 1:**
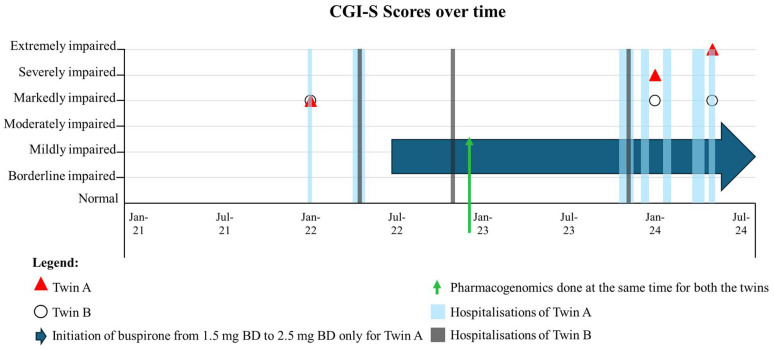
Clinical history of twin A and B. A: RTT-anchored Clinical Global Impression Scale for Severity (RTT-CGI-S) scores over time.

**Figure 2:**
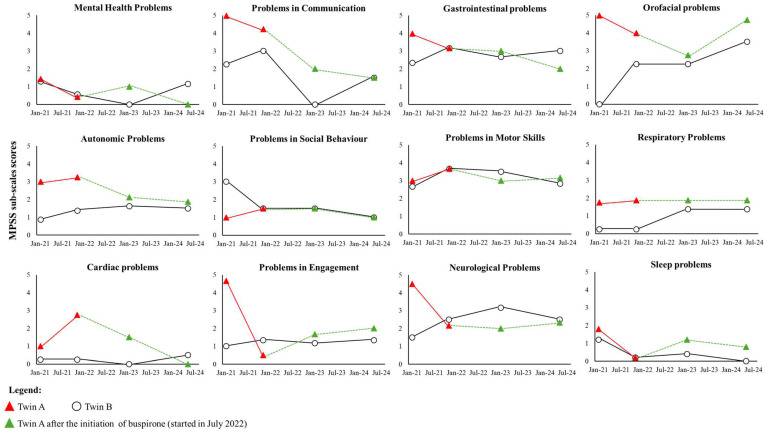
Multi-System Profile of Symptoms Scale (MPSS) scores over time.

Hospitalisations were used as a proxy for clinical severity. This showed that twin A had more than double the number of hospitalisations (*n* = 7) than twin B (*n* = 3). The duration of hospital stays was also more pronounced for twin A. Twin A stayed for up to 5 weeks in hospital while twin B stayed for no more than a week, despite both being admitted for the same reason. The RTT-anchored CGI-S ratings for twins A and B were markedly different, with twin A showing symptom deterioration reflected by a greater degree of impairment over time (Figure 1). In comparison, twin B remained clinically stable over the same time epoch. When examining the symptom frequency using the MPSS, the MPSS sub-scale scores for communication, orofacial, autonomic, respiratory, and cardiac problems were higher for twin A when compared to twin B (Figure 2).

#### 3.1.1. Initiation of Buspirone (Twin A)

Twin A was initiated (started in July 2022) on buspirone due to the severity of autonomic symptoms. Buspirone was initiated at 1.5 mg BD and titrated up to 2.5 mg BD, and it improved symptoms related to autonomic and cardiac problems, with the MPSS sub-scale scores returning to values seen for twin B. Buspirone also appeared to stabilise symptoms related to communication and neurological problems (Figure 2).

#### 3.1.2. Bayesian Inferential Methods: Frequentist Significance Test

When comparing the MPSS sub-scale scores for twin A and B with the RTT reference population (n = 106) used to validate MPSS [34], the results showed that the profile of symptoms varies across the different time points for both twins (Supplementary Information S2). Specifically, the mental health problems sub-scale scores for twin A progressed from being in line with the reference RTT population to a less frequent deficit. Twin B showed a similar profile. The items which investigated the autonomic problems showed that twin A moved from a more frequent presence of the symptoms to being in line with the overall population. Similarly, twin B fluctuated between a less frequent presence of the symptoms to alignment with the reference population. The cardiac problems for twin A were noted to improve over time. On the contrary, the cardiac symptoms for twin B became worse but were in line with the reference population. Problems in communication and in engagement for twin A became less frequent over time; however, the symptoms for twin B were more stable. Both the twins presented with progressively less frequent problems in social behaviour. Gastrointestinal profiles were different for the twins. Problems in motor skills for both the twins fluctuated between less frequent to being in line with the reference population. The neurological and orofacial problems became significantly worse only for twin B. The respiratory problems of twin A evolved to a slightly more severe profile with respect to twin B. Finally, the frequency of the sleep disturbances of twin A showed an initial improvement that then realigned with the reference population, while twin B improved from having less severe to less frequent symptoms.

### 3.2. Longitudinal Assessment of HRV Parameters

**Figure 3:**
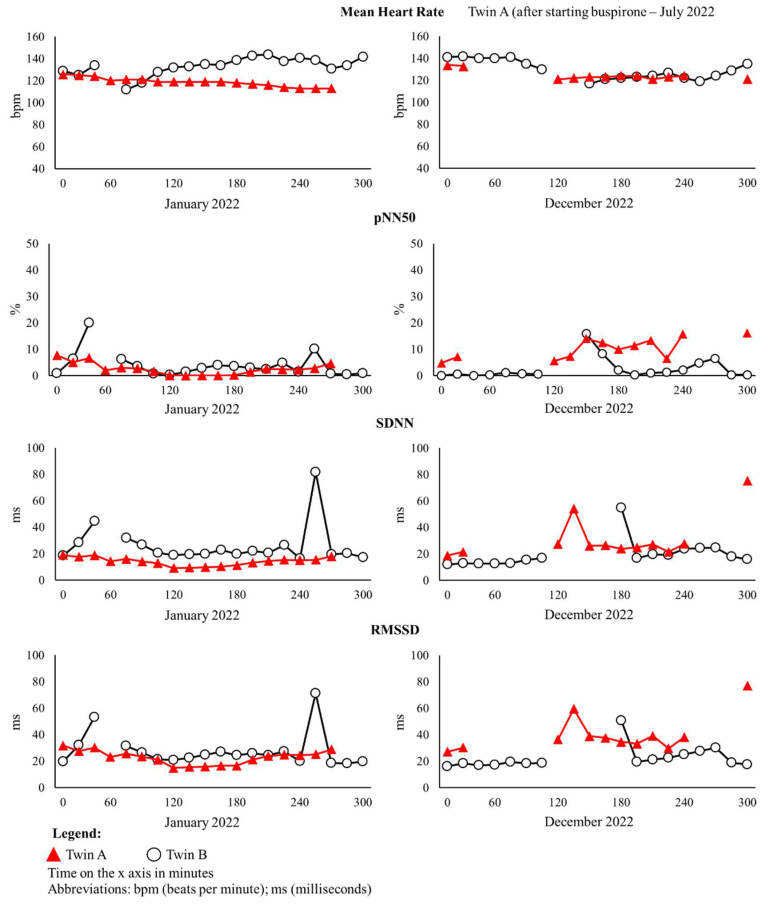
Longitudinal assessment of heart rate variability parameters.

Mean HR values were similar for both twins A and B. Autonomic metrics for twin A were low during January 2022. However, autonomic health for twin B was relatively stable across the 12 months, as reflected by similar RMSSD and SDNN values between awake and sleep periods during this time (Table 1). Longitudinal assessment over a 5 h awake period also showed low values for parasympathetic indices (pNN50 and RMSSD) and the sympathetic index (SDNN) (Figure 3).

#### 3.2.1. After Initiation of Buspirone (Twin A)

After the initiation of buspirone (July 2022), autonomic metrics all increased for both the awake and sleep cycle across 12 months (Table 1). These findings are further corroborated over a 5 h awake period that showed an improvement in autonomic metrics (pNN50, RMSSD, and SDNN) following the initiation of buspirone (Figure 3 and Supplementary Information S3).

#### 3.2.2. Non-Parametric Wilcoxon Signed-Rank Tests

The results of the Wilcoxon signed-rank tests showed that there were significant differences between twins A and B for mean HR (Z = −3.260; *p* = 0.001) and SDNN (Z = −3.524; *p* < 0.001) in data collected in January 2022, with twin A having lower values for both HR and SDNN. Similarly, we found that twin B presented with a lower pNN50 in December 2022 with respect to twin A (Z = −2.703; *p* = 0.007). For twin A, all the HRV parameters showed a significant difference between January and December 2022: mean HR (Z = −2.937; *p* = 0.003), pNN50 (Z = −2.763; *p* = 0.006), SDNN (Z = −2.807; *p* = 0.005), and RMSSD (Z = −2.714; *p* = 0.007).

### 3.3. MECP2 and BDNF Genotyping

Table 2 shows the *MECP2* and *BDNF* genotypes for twins A and B. Both twins had identical pathogenic *MECP2* mutations; however, twin A also had a co-occurring rs6265 *BDNF* polymorphism predicted to cause impaired BDNF function.

#### 3.3.1. *MECP2*

The genotyping results for twins A and B confirmed a deletion in *MECP2* (NM_004992.3). This was a 5.5 kb deletion that resulted in the loss of 100 residues of the protein (including the canonical stop codon), followed by the insertion of a glutamine residue and a new stop codon within 3′UTR (3′ untranslated region) (PM4 [protein length change] moderate) (see Supplementary Information S4).

#### 3.3.2. *BDNF*

The *BDNF* genotyping results confirmed the following: Twin A had the *BDNF 434C>T C/T* genotype (heterozygous for rs6265 T allele). This rs6265 polymorphism, also known as G196A or Val66Met, is caused by a single nucleotide substitution (guanine to adenine) at position 196 of the *BDNF* gene. This substitution causes a change from valine (Val) to methionine (Met) at codon 66 (Val66Met) of the N-terminal pro-domain of the pre-cursor BDNF protein [46]. The *rs6265* polymorphism is associated with reduced activity-dependent secretion of BDNF from neurons and impaired BDNF signalling [47]. Twin B had the *BDNF 434C>T C/C* genotype (homozygous for *rs6265* C allele), which is predicted to result in normal activity-dependent secretion of BDNF from neurons and normal BDNF signalling.

## 4. Discussion

Our case report used a precision medicine approach to better understand the discordant neurodevelopment phenotypes in twins with RTT having identical pathogenic *MECP2* mutations. For the first time, we have demonstrated that a triumvirate of targeted genotyping, longitudinal objective physiological monitoring, and measures of clinical severity in RTT patients can help disentangle clinical outcomes, thereby assisting in clinical risk stratification. We show that twin A has (I) a co-occurring *BNDF* mutation, (II) less autonomic flexibility, and (III) poorer clinical outcomes than twin B. These findings are important because they can be extended to other neurodevelopmental disorders to understand clinical outcomes, especially in more clinically vulnerable patients with unclear genotype–phenotype associations.

Several outcome measures have been used to assess the clinical severity of RTT. The RTT-CGI has been used in studies of RTT [48,49,50], although some have suggested that the RTT-CGI could be difficult to implement when used more widely, especially when knowledge of RTT is limited [51]. In the current case report, the RTT-CGI-S showed that twin A was markedly more impaired than twin B. When honing into the troublesome symptoms, the MPSS revealed that communication, orofacial, autonomic, respiratory, and cardiac problems were more prominent for twin A. Twin A had poor autonomic and cardiac health, as identified by symptom profiling, severity, and HRV parameters. This improved after the initiation of buspirone. The results from the Bayesian inferential methods test also showed that the profile of RTT patients can change over time, with some symptoms improving while others may worsen. Regression is variable in RTT and, hence, the changes following buspirone treatment might not be so obvious. When viewed together, our findings highlight the importance of using an outcome measure to capture symptom change across several clinical domains alongside objective physiological data when assessing clinical severity in RTT and other disorders.

Autonomic dysregulation in RTT has been characterised in the literature and is not the focus of the current case report. However, we can further enrich the evidence base by examining a twin with identical *MECP2* mutations. In our case report, the measurement of the PNS using RMSSD [35,36,52,53] and SNS using SDNN [54] demonstrated that twin A had poorer autonomic health than twin B, as indicated by the depressed autonomic metrics. Twin A (pre-buspirone treatment) had autonomic inflexibility and was less likely to adapt to stimuli from demands imposed by the SNS and PNS from concomitant autonomic stressors frequent in RTT, such as pro-arrhythmic (QT prolongation) medications, seizures, infections/sepsis, and medications that cause respiratory depression. Following buspirone treatment, there were statistically significant increases in all HRV parameters for twin A. Patients with RTT are at higher risk of Sudden Unexpected Death in Epilepsy (SUDEP) [55], and RMSSD forms part of the SUDEP-7 risk inventory with lower RMSSD scores indicating a risk factor for SUDEP [52]. The initiation of buspirone shifted twin A from an inflexible to a more flexible autonomic profile, resulting in a profile more likely to adapt to autonomic stressors. This finding is also mirrored in our MPSS data, which showed a reduction in autonomic and cardiac symptoms following buspirone treatment. When viewed together, although twin A has clinically more RTT severity than twin B, the initiation of buspirone provided a safety net for twin A by preventing the autonomic and cardiac symptoms from becoming worse. When examining the lens of other neurodevelopmental disorders, a meta-analysis has suggested that low HRV and poor vagal control are potential biomarkers in autism spectrum disorder (ASD) [56]. It could therefore be surmised that a depressed autonomic imbalance in twin A could be a predictor of poorer neurodevelopmental outcomes, which were in part remedied by buspirone treatment. A recent study suggests autonomic dysregulation during very early infancy could play a key role in the underlying features of ASD symptomatology [57] and could add credence to this hypothesis.

Buspirone is a partial agonist that has a high affinity for 5-HT_1A_ receptors [58] and a D2 receptor antagonist [59]. It has been used for managing the symptoms seen in neuropsychiatric disorders, spinocerebellar ataxias, and tardive dyskinesia in Parkinson’s disease [59]. It may also be helpful for treating behavioural dysregulation following brain injury [60], blunting central apnoea’s and improving oxygen saturation in patients with heart failure [61], and remission of apneusis in a child with an astrocytoma affecting the respiratory centres of the brain [62]. Some previous reports have shown improvements in respiratory dysfunction following buspirone treatment in RTT patients [63,64,65]. Other 5-HT_1A_ agonists, such as tandospirone, have also been shown to reduce sleep apnoeic events in an 11-year-old girl with RTT [66]. Another study showed mixed improvement following buspirone treatment, compared to topiramate and acetazolamide, for managing irregular breathing in RTT patients [67]. Serotonergic (5-hydroxytryptamine, 5-HT) 5-HT_1A_ receptors have an important role in anxiety and depression [68]. Buspirone augmentation may help in patients with severe depression [69]. Indeed, selective serotonin reuptake inhibitors such as buspirone have been used for managing anxiety and other behaviour disorders in RTT patients [70,71]. In RTT, buspirone can also improve the symptoms of Emotional, Behavioural, and Autonomic Dysregulation (EBAD) [20]. When viewed together, buspirone seems a promising drug to manage the symptoms of EBAD, such as autonomic dysregulation, breathing problems. or anxiety in RTT.

The precise mechanism by which buspirone might improve autonomic flexibility in RTT patients is unknown. The underlying features of EBAD and the associated autonomic dysregulation are complex, and they are therefore unlikely to result from a single unified mechanism. In RTT, evidence points towards a central defect in monoaminergic systems [72], and we have suggested previously that defects in serotonergic neurotransmitter systems could be one aspect of better understanding the broad clinical phenotypes in RTT [73]. As proposed by others [61], acting through pre-synaptic 5-HT_1A_ auto-receptors, buspirone modulates the amount of serotonin released from the synaptic cleft. The serotonergic network innervates into the dorsal brainstem, which contains areas critical for cardio-respiratory control, such as the Kölliker–Fuse and solitary tract nuclei [72,73]. The action of 5-HT on breathing is dependent upon both pre-synaptic and post-synaptic 5-HT neurons associated with respiratory function, and it is suggested that the increase in the firing rate of 5-HT neurons leads to an overall net facilitatory effect on respiration [74]. In RTT animal models, 5-HT1A agonists have been shown to reverse the deleterious respiratory phenotype [75].

Buspirone might help regulate the altered serotonergic tone in RTT and improve the functioning of 5-HT neuronal networks. This premise might be more critical for RTT patients with co-occurring *BDNF* polymorphisms because evidence has shown that BDNF is crucial for the proper functioning of the serotonergic neurotransmitter system [76] and is dysregulated in Kölliker–Fuse nuclei in infants with unexplained sudden death [77]. We have suggested that BDNF dysregulation disrupts the neuronal firing in critical regions of the brainstem in RTT, which are important for cardiorespiratory autonomic function [73]. By strengthening post-synaptic inhibition, the net effect of buspirone in RTT could be used to suppress the activation of respiratory neurons, which in turn would normalise the dysregulated inspiratory and expiratory neurons [66] in the brainstem and improve breathing. Buspirone might therefore help to limit the autonomic stress on an already weakened autonomic system, as was seen for twin A, which had a co-occurring *rs6265* BDNF polymorphism that is associated with a reduced secretion of BDNF from neurons and impaired BDNF signalling [47,78]. By regulating autonomic stress, buspirone could therefore safeguard against the triggers that cause EBAD to worsen (such as deteriorating cardiac and respiratory symptoms). Buspirone as an adjunct with other medication(s) might therefore be sufficient to impact the quality of life and/or improve the long-term outcomes for RTT patients at higher risk of EBAD. However, this case report involves only one pair of twins, which limits the generalizability of the findings, and this hypothesis would need to be evaluated in a much larger sample.

Brain development is dependent on *BDNF* function [79]. In RTT, *BDNF* dysregulation has previously been described [80]; however, it is still unclear how *MECP2* might regulate *BDNF* expression in RTT. Transcript levels of *BDNF* vary in the brain of RTT patients [81], and post-mortem brain samples from four RTT patients have shown no discernible differences in BDNF immunolabelling compared to controls [82]. While animal models are helpful, they are unable to provide a robust framework for *MECP2* and *BDNF* regulation in patients. Earlier evidence has indicated that *BDNF* is a gene target for *MECP2* [83]. The *rs6265 BDNF* polymorphism seems to be a common polymorphism in RTT [84] and is suggested to be a disease modifier in RTT patients [84,85], although there is some conflicting evidence [86]. More recently, a bioinformatic meta-analysis using weighted gene correlation network analysis (WGCNA) examined dysregulated genes across an enriched data set and showed that *BDNF* is downregulated in RTT [87]. Because brain development depends on BDNF function, it is not surprising that *BDNF* impairment is implicated in some of the psychopathology observed in different neurodevelopmental disorders [79]. Indeed, some evidence also suggests a link between *BDNF* polymorphisms and an increased risk for anxiety and attention deficit hyperactivity disorder (ADHD) [79]. It is therefore entirely plausible that in the presence of an identical pathogenic *MECP2* mutation, a co-occurring *rs6265 BDNF* polymorphism that negatively impacts upon BDNF secretion and signalling could lead to a more deleterious impact on brain functioning and, hence, poorer clinical outcomes for twin A.

Individualised care is vital for the wellbeing of patients with RTT, and enriched environments can help to improve outcomes in some neurodevelopmental disorders [88]. Twin A spent more time in hospital, and it is likely that the associated consequences of hospitalisation for twin A are a driver for worse clinical outcomes. In RTT, environmental enrichment can improve functional outcomes, possibly through the strengthening of neuronal pathways dependent upon BDNF function [84]. Our previous evidence synthesis has also indicated that sensory issues can worsen the autonomic profile in RTT [89]. When taken together, environmental factors should be considered when examining the divergent clinical profiles of twins A and B.

## 5. Conclusions

This case report illustrates the impact of precision medicine on the assessment of neurodevelopmental outcomes in RTT. When disease-related genotype–phenotype prognosticators are limited, a multimodal approach that incorporates targeted genotyping and longitudinal physiological monitoring can help disentangle complex clinical symptoms and severity profiles in RTT. We showed that a combination of a co-occurring *rs6265 BDNF* polymorphism and autonomic inflexibility led to a poorer clinical outcome for twin A. Using buspirone, we were also able to shift a rigid autonomic profile to a more flexible one for twin A and thereby prevent the cardiac and autonomic symptoms from worsening. In RTT, *BDNF* is downregulated [87]. While this case report does not explore the ramifications of *BDNF* downregulation and its neurobiological underpinnings, *BDNF* is likely pleiotropic and affects phenotypic traits dependent on environmental and mental health factors. Therefore, co-occurring *BDNF* impairment in RTT could have a far-reaching impact on clinical phenotypes and clinical outcomes.

In summary, precision medicine that uses a triumvirate of targeted genotyping, longitudinal objective physiological monitoring, and measures of clinical severity have allowed us to unravel discordant symptom profiles in twins with RTT with an identical pathogenic *MECP2* mutation. There could also be a case for a more streamlined approach to developing targeted genotype panels, as previously suggested [90]. Targeted genotyping of RTT patients would be quicker and more cost-effective. These aspects are important given that in the UK, barriers to implement genotyping in mental healthcare include cost, lack of knowledge about genotyping, and concerns about how it could be incorporated into the current clinical care workflows [91].

## 6. Limitations

Our precision medicine approach, while still in its infancy, can be extended to other areas and would allow clinicians to make informed choices regarding the severity of the illness and the treatment pathway, ultimately leading to improvements in patient care. Nevertheless, this approach must be validated in a larger data set. While the assessment of HRV parameters suggests autonomic inflexibility in twin A compared to twin B, it would be premature to suggest that this pattern would be relevant to the broader RTT population. A much larger sample using biometric sensor-based physiological monitoring would be required to explore the magnitude of awake and sleep longitudinal autonomic disturbances in RTT and how they might influence on clinical outcomes. While twin A had a higher frequency of problems across different symptom domains, symptom frequency using the MPSS would also need to be examined alongside autonomic HRV parameters in a larger sample to better understand group data regarding HRV parameters and clinical severity profiles. The zygosity of the twins was reported in the medical notes by parents. However, we were unable to confirm the zygosity of the twins genetically.

## Figures and Tables

**Table 1:** Heart rate variability parameters.

		Twin A		Twin B	
Metric	Epoch	Awake	Sleep	Awake	Sleep
Mean (bpm) HR (min; max)	01/2022	119 (38; 175)	133 (70; 160)	130 (67; 175)	129 (82; 175)
	12/2022	125 (59; 160)	128 (72; 167)	126 (57; 175)	131 (98; 160)
pNN50 (%)	01/2022	3.33	0.18	3.34	0.534
	12/2022	11.89	1.24	1.48	0.1
SDNN (ms)	01/2022	29.9	12.05	42.19	21.74
	12/2022	39.41	21.28	42.24	24.35
RMSSD (ms)	01/2022	28.76	16.61	25.89	17.91
	12/2022	44.44	19.60	25.77	13.39

**Table 2:** Genotyping of *MECP2 and BDNF* for twin A and B.

	*MECP2*		*BDNF*	
	Genotype *	Phenotype	Genotype	Phenotype
Twin A	Heterozygous c.1160_*5215del p(Pro387_Ser486delinsGln)	Pathogenic	*434C>T C/T* (heterozygous for rs6265 T Allele)	Associated with reduced activity-dependent secretion of BDNF from neurons and impaired BDNF signalling
Twin B	Heterozygous c.1160_*5215del p(Pro387_Ser486delinsGln)	Pathogenic	*434C>T C/C* (homozygous for rs6265 C Allele)	Associated with normal activity-dependent secretion of BDNF from neurons and normal BDNF signalling

* protein information is also shown. Abbreviations: *BDNF* (gene that encodes brain-derived neurotrophic factor protein), *MECP2* (gene that encodes methyl CpG binding protein 2).

## Data Availability

Data can be obtained upon reasonable request from the corresponding author.

## Supplementary Materials

The following supporting information can be downloaded at: https://www.mdpi.com/article/10.3390/cimb46080497/s1, Supplementary Information S1: Duration of Awake and Sleep Periods. Supplementary Information S2: MPSS—Bayesian inferential methods: frequentist significance test. Supplementary Information S3: Longitudinal Assessment of Heart Rate Variability Parameters for Twin A (pre and post buspirone initiation). Supplementary Information S4: Genotype Results.
