# Supplementary material for: Using Precision Medicine to Disentangle Genotype–Phenotype Relationships in Twins with Rett Syndrome: A Case Report
Source: Curr Issues Mol Biol. 2024 Aug 2;46(8):8424–40. doi: 10.3390/cimb46080497 (PMC11352978; doi:10.3390/cimb46080497)
Supplement: Supplementary file 1 [file cimb-46-00497-s001.zip › cimb-3101672-supplementary.pdf]

Supplementary Information - Contents

Supplementary Information S1: Duration of Awake and Sleep Periods

Supplementary Information S2: MPSS - Bayesian inferential methods: frequentist significance test

Supplementary Information S3: Longitudinal Assessment of Heart Rate Variability Parameters for Twin A (pre and post buspirone initiation)

Supplementary Information S4: Genotype Results

Supplementary Information S1: Duration of Awake and Sleep Periods

	Type of Activity	Start time	End time	Total time
Twin A	Awake	09/01/2022 - 16:00:00	09/01/2022 - 22:00:00	06:00:00
	Asleep	09/01/2022 - 00:00:00	09/01/2022 - 05:00:00	05:00:00
	Awake	09/12/2022 - 16:00:00	09/12/2022 - 21:00:00	05:00:00
	Asleep	09/12/2022 - 00:00:00	09/12/2022 - 06:00:00	06:00:00
Twin B	Awake	22/01/2022 - 14:00:00	22/01/2022 - 21:00:00	07:00:00
	Asleep	22/01/2022 - 00:00:00	22/01/2022 - 06:00:00	06:00:00
	Awake	01/12/2022 - 10:00:00	01/12/2022 - 21:00:00	11:00:00
	Asleep	01/12/2022 - 00:00:00	01/12/2022 - 05:00:00	05:00:00

Supplementary Information S2: MPSS - Bayesian inferential methods: frequentist significance test

Mental Health Problems									
Population (n=106)		Test statistics							
Mean	Standard deviation			Individual's test score	Probability		Estimate of percentage of control population falling below patient's score		
1.399	1.312	Twin A	TP1	1.429	One-tailed	0.491	Lower	43.35%
					Two-tailed	0.982	Upper	58.43%
							Bayesian point	50.90%
			TP2	0.429	One-tailed	0.232	Lower	17.04%
					Two-tailed	0.463	Upper	30.05%
							Bayesian point	23.17%
			TP3	1.000	One-tailed	0.381	Lower	30.97%
					Two-tailed	0.763	Upper	45.70%
							Bayesian point	38.14%
			TP4	0.000	One-tailed	0.145	Lower	9.63%
					Two-tailed	0.291	Upper	20.46%
							Bayesian point	14.55%
		Twin B	TP1	1.286	One-tailed	0.466	Lower	39.10%
					Two-tailed	0.932	Upper	54.11%
							Bayesian point	46.59%
			TP2	0.571	One-tailed	0.266	Lower	20.14%
					Two-tailed	0.531	Upper	33.68%
							Bayesian point	26.56%
			TP3	0.000	One-tailed	0.146	Lower	9.65%
					Two-tailed	0.291	Upper	20.44%
							Bayesian point	14.56%
			TP4	1.143	One-tailed	0.423	Lower	35.01%
					Two-tailed	0.846	Upper	49.87%
							Bayesian point	42.32%

Autonomic Problems								
Population (n=106)				Test statistics				
Mean	Standard deviation			Individual's test score	Probability		Estimate of percentage of control population falling below patient's score	
1.348	0.973	**Twin A**	TP1	3.000	One-tailed	0.047	Lower	91.86%
					Two-tailed	0.094	Upper	97.70%
							Bayesian point	95.29%
			TP2	3.250	One-tailed	0.027	Lower	94.80%
					Two-tailed	0.054	Upper	98.86%
							Bayesian point	97.28%
			TP3	2.125	One-tailed	0.214	Lower	71.87%
					Two-tailed	0.428	Upper	84.50%
							Bayesian point	78.58%
			TP4	1.875	One-tailed	0.295	Lower	63.16%
					Two-tailed	0.591	Upper	77.18%
							Bayesian point	70.45%
		**Twin B**	TP1	0.875	One-tailed	0.315	Lower	24.64%
					Two-tailed	0.630	Upper	38.78%
							Bayesian point	31.48%
			TP2	1.375	One-tailed	0.489	Lower	43.60%
					Two-tailed	0.978	Upper	58.62%
							Bayesian point	51.10%
			TP3	1.625	One-tailed	0.389	Lower	53.64%
					Two-tailed	0.777	Upper	68.42%
							Bayesian point	61.14%
			TP4	1.500	One-tailed	0.438	Lower	48.62%
					Two-tailed	0.877	Upper	63.54%
							Bayesian point	56.16%

Cardiac Problems									
Population (n=106)		Test statistics							
Mean	Standard deviation			Individual's test score	Probability		Estimate of percentage of control population falling below patient's score		
0.636	0.937	**Twin A**	TP1	1.000	One-tailed	0.350	Lower	57.51%	
					Two-tailed	0.700	Upper	72.07%	
							Bayesian point	65.01%	
			TP2	2.750	One-tailed	0.013	Lower	97.10%	
					Two-tailed	0.027	Upper	99.55%	
							Bayesian point	98.66%	
			TP3	1.500	One-tailed	0.180	Lower	75.55%	
					Two-tailed	0.361	Upper	87.42%	
							Bayesian point	81.96%	
			TP4	0.000	One-tailed	0.250	Lower	18.72%	
					Two-tailed	0.501	Upper	32.07%	
							Bayesian point	25.03%	
		**Twin B**	TP1	0.250	One-tailed	0.341	Lower	27.13%	
					Two-tailed	0.683	Upper	41.60%	
							Bayesian point	34.14%	
			TP2	0.250	One-tailed	0.341	Lower	27.11%	
					Two-tailed	0.683	Upper	41.56%	
							Bayesian point	34.13%	
			TP3	0.000	One-tailed	0.250	Lower	18.72%	
					Two-tailed	0.501	Upper	32.07%	
							Bayesian point	25.05%	
			TP4	0.000	One-tailed	0.250	Lower	18.74%	
					Two-tailed	0.501	Upper	32.04%	
							Bayesian point	25.05%	

Problems in Communication								
Population (n=106)		Test statistics						
Mean	Standard deviation			Individual's test score	Probability		Estimate of percentage of control population falling below patient's score	
3.58	1.377	Twin A	TP1	5.000	One-tailed	0.153	Lower	78.65%
					Two-tailed	0.307	Upper	89.74%
							Bayesian point	84.66%
			TP2	4.250	One-tailed	0.315	Lower	61.20%
					Two-tailed	0.629	Upper	75.38%
							Bayesian point	68.54%
			TP3	2.000	One-tailed	0.128	Lower	8.20%
					Two-tailed	0.256	Upper	18.39%
							Bayesian point	12.80%
			TP4	1.500	One-tailed	0.068	Lower	3.68%
					Two-tailed	0.136	Upper	10.99%
							Bayesian point	6.78%
		Twin B	TP1	2.250	One-tailed	0.169	Lower	11.60%
					Two-tailed	0.339	Upper	23.19%
							Bayesian point	16.93%
			TP2	3.000	One-tailed	0.338	Lower	26.82%
					Two-tailed	0.676	Upper	41.26%
							Bayesian point	33.79%
			TP3	0.000	One-tailed	0.006	Lower	0.14%
					Two-tailed	0.011	Upper	1.40%
							Bayesian point	0.55%
			TP4	1.500	One-tailed	0.068	Lower	3.70%
					Two-tailed	0.136	Upper	11.00%
							Bayesian point	6.79%

Problems in Social Behaviour									
Population (n=106)		Test statistics							
Mean	Standard deviation			Individual's test score	Probability		Estimate of percentage of control population falling below patient's score		
1.835	1.492	Twin A	TP1	1.000	One-tailed	0.289	Lower	22.28%
					Two-tailed	0.578	Upper	36.16%
							Bayesian point	28.92%
			TP2	1.500	One-tailed	0.412	Lower	33.89%
					Two-tailed	0.824	Upper	48.80%
							Bayesian point	41.19%
			TP3	1.500	One-tailed	0.412	Lower	33.86%
					Two-tailed	0.824	Upper	48.72%
							Bayesian point	41.18%
			TP4	1.000	One-tailed	0.289	Lower	22.27%
					Two-tailed	0.579	Upper	36.22%
							Bayesian point	28.94%
		Twin B	TP1	3.000	One-tailed	0.220	Lower	71.28%
					Two-tailed	0.439	Upper	84.06%
							Bayesian point	78.05%
			TP2	1.500	One-tailed	0.412	Lower	33.87%
					Two-tailed	0.824	Upper	48.79%
							Bayesian point	41.20%
			TP3	1.500	One-tailed	0.412	Lower	33.85%
					Two-tailed	0.824	Upper	48.79%
							Bayesian point	41.18%
			TP4	1.000	One-tailed	0.289	Lower	22.26%
					Two-tailed	0.579	Upper	36.18%
							Bayesian point	28.93%

Problems in Engagement									
Population (n=106)		Test statistics							
Mean	Standard deviation			Individual's test score	Probability		Estimate of percentage of control population falling below patient's score		
1.643	1.372	Twin A	TP1	4.667	One-tailed	0.015	Lower	96.77%
					Two-tailed	0.030	Upper	99.47%
							Bayesian point	98.48%
			TP2	0.500	One-tailed	0.204	Lower	14.59%
					Two-tailed	0.409	Upper	27.07%
							Bayesian point	20.44%
			TP3	1.667	One-tailed	0.493	Lower	43.08%
					Two-tailed	0.986	Upper	58.23%
							Bayesian point	50.69%
			TP4	2.000	One-tailed	0.398	Lower	52.62%
					Two-tailed	0.796	Upper	67.47%
							Bayesian point	60.18%
		Twin B	TP1	1.000	One-tailed	0.321	Lower	25.26%
					Two-tailed	0.642	Upper	39.42%
							Bayesian point	32.10%
			TP2	1.333	One-tailed	0.411	Lower	33.78%
					Two-tailed	0.822	Upper	48.69%
							Bayesian point	41.12%
			TP3	1.167	One-tailed	0.365	Lower	29.35%
					Two-tailed	0.731	Upper	44.01%
							Bayesian point	36.53%
			TP4	1.333	One-tailed	0.412	Lower	33.80%
					Two-tailed	0.823	Upper	48.78%
							Bayesian point	41.16%

Gastrointestinal Problems									
Population (n=106)		Test statistics							
Mean	Standard deviation			Individual's test score	Probability		Estimate of percentage of control population falling below patient's score		
2.107	1.442	Twin A	TP1	4.000	One-tailed	0.097	Lower	85.33%
					Two-tailed	0.194	Upper	94.18%
							Bayesian point	90.29%
			TP2	3.167	One-tailed	0.233	Lower	69.83%
					Two-tailed	0.466	Upper	82.87%
							Bayesian point	76.71%
			TP3	3.000	One-tailed	0.269	Lower	65.96%
					Two-tailed	0.539	Upper	79.56%
							Bayesian point	73.07%
			TP4	2.000	One-tailed	0.471	Lower	39.57%
					Two-tailed	0.941	Upper	54.66%
							Bayesian point	47.06%
		Twin B	TP1	2.333	One-tailed	0.438	Lower	48.66%
					Two-tailed	0.876	Upper	63.60%
							Bayesian point	56.19%
			TP2	3.167	One-tailed	0.233	Lower	69.83%
					Two-tailed	0.466	Upper	82.85%
							Bayesian point	76.70%
			TP3	2.667	One-tailed	0.350	Lower	57.49%
					Two-tailed	0.700	Upper	72.06%
							Bayesian point	65.00%
			TP4	3.000	One-tailed	0.269	Lower	65.94%
					Two-tailed	0.539	Upper	79.56%
							Bayesian point	73.05%

Problems in Motor Skills									
Population (n=106)		Test statistics							
Mean	Standard deviation			Individual's test score	Probability		Estimate of percentage of control population falling below patient's score		
3.679	1.252	Twin A	TP1	3.000	One-tailed	0.295	Lower	22.81%
					Two-tailed	0.590	Upper	36.79%
							Bayesian point	29.52%
			TP2	3.667	One-tailed	0.496	Lower	42.06%
					Two-tailed	0.993	Upper	57.24%
							Bayesian point	49.63%
			TP3	3.000	One-tailed	0.295	Lower	22.81%
					Two-tailed	0.590	Upper	36.80%
							Bayesian point	29.51%
			TP4	3.167	One-tailed	0.343	Lower	27.21%
					Two-tailed	0.685	Upper	41.69%
							Bayesian point	34.25%
		Twin B	TP1	2.667	One-tailed	0.212	Lower	15.23%
					Two-tailed	0.423	Upper	27.89%
							Bayesian point	21.16%
			TP2	3.667	One-tailed	0.496	Lower	42.09%
					Two-tailed	0.992	Upper	57.17%
							Bayesian point	49.61%
			TP3	3.500	One-tailed	0.444	Lower	36.93%
					Two-tailed	0.887	Upper	51.91%
							Bayesian point	44.36%
			TP4	2.883	One-tailed	0.264	Lower	19.96%
					Two-tailed	0.528	Upper	33.49%
							Bayesian point	26.39%

Neurological Problems									
Population (n=106)		Test statistics							
Mean	Standard deviation			Individual's test score	Probability		Estimate of percentage of control population falling below patient's score		
1.847	1.334	Twin A	TP1	4.500	One-tailed	0.025	Lower	95.14%	
					Two-tailed	0.050	Upper	98.97%	
							Bayesian point	97.48%	
			TP2	2.167	One-tailed	0.406	Lower	51.86%	
					Two-tailed	0.812	Upper	66.73%	
							Bayesian point	59.40%	
			TP3	2.000	One-tailed	0.455	Lower	46.93%	
					Two-tailed	0.909	Upper	62.06%	
							Bayesian point	54.55%	
			TP4	2.333	One-tailed	0.359	Lower	56.66%	
					Two-tailed	0.718	Upper	71.21%	
							Bayesian point	64.12%	
		Twin B	TP1	1.500	One-tailed	0.398	Lower	32.57%	
					Two-tailed	0.796	Upper	47.34%	
							Bayesian point	39.81%	
			TP2	2.500	One-tailed	0.314	Lower	61.27%	
					Two-tailed	0.627	Upper	75.50%	
							Bayesian point	68.63%	
			TP3	3.167	One-tailed	0.163	Lower	77.52%	
					Two-tailed	0.327	Upper	88.88%	
							Bayesian point	83.67%	
			TP4	2.500	One-tailed	0.314	Lower	61.31%	
					Two-tailed	0.627	Upper	75.51%	
							Bayesian point	68.65%	

Orofacial Problems									
Population (n=106)		Test statistics							
Mean	Standard deviation			Individual's test score	Probability		Estimate of percentage of control population falling below patient's score		
1.715	1.543	Twin A	TP1	5.000	One-tailed	0.018	Lower	96.27%
					Two-tailed	0.036	Upper	99.33%
							Bayesian point	98.18%
			TP2	4.000	One-tailed	0.072	Lower	88.53%
					Two-tailed	0.143	Upper	96.05%
							Bayesian point	92.83%
			TP3	2.750	One-tailed	0.253	Lower	67.71%
					Two-tailed	0.506	Upper	81.12%
							Bayesian point	74.72%
			TP4	4.750	One-tailed	0.026	Lower	94.96%
					Two-tailed	0.053	Upper	98.91%
							Bayesian point	97.36%
		Twin B	TP1	0.000	One-tailed	0.136	Lower	8.79%
					Two-tailed	0.271	Upper	19.30%
							Bayesian point	13.56%
			TP2	2.250	One-tailed	0.365	Lower	55.99%
					Two-tailed	0.730	Upper	70.61%
							Bayesian point	63.45%
			TP3	2.250	One-tailed	0.365	Lower	55.95%
					Two-tailed	0.731	Upper	70.63%
							Bayesian point	63.48%
			TP4	3.500	One-tailed	0.126	Lower	81.84%
					Two-tailed	0.252	Upper	91.94%
							Bayesian point	87.40%

Respiratory Problems									
Population (n=106)		Test statistics							
Mean	Standard deviation			Individual's test score	Probability		Estimate of percentage of control population falling below patient's score		
1.360	1.138	Twin A	TP1	1.750	One-tailed	0.367	Lower	55.82%
					Two-tailed	0.734	Upper	70.46%
							Bayesian point	63.32%
			TP2	1.875	One-tailed	0.327	Lower	59.85%
					Two-tailed	0.654	Upper	74.24%
							Bayesian point	67.31%
			TP3	1.875	One-tailed	0.327	Lower	59.95%
					Two-tailed	0.653	Upper	74.26%
							Bayesian point	67.33%
			TP4	1.875	One-tailed	0.327	Lower	59.90%
					Two-tailed	0.653	Upper	74.28%
							Bayesian point	67.33%
		Twin B	TP1	0.250	One-tailed	0.167	Lower	11.40%
					Two-tailed	0.334	Upper	22.90%
							Bayesian point	16.71%
			TP2	0.250	One-tailed	0.167	Lower	11.40%
					Two-tailed	0.334	Upper	22.90%
							Bayesian point	16.69%
			TP3	1.375	One-tailed	0.495	Lower	42.99%
					Two-tailed	0.990	Upper	58.09%
							Bayesian point	50.52%
			TP4	1.375	One-tailed	0.495	Lower	42.99%
					Two-tailed	0.990	Upper	58.04%
							Bayesian point	50.50%

Sleep Problems									
Population (n=106)		Test statistics							
Mean	Standard deviation			Individual's test score	Probability		Estimate of percentage of control population falling below patient's score		
1.034	0.993	**Twin A**	TP1	1.800	One-tailed	0.222	Lower	70.96%	
					Two-tailed	0.444	Upper	83.83%	
							Bayesian point	77.79%	
			TP2	0.200	One-tailed	0.203	Lower	14.46%	
					Two-tailed	0.405	Upper	26.88%	
							Bayesian point	20.26%	
			TP3	1.200	One-tailed	0.434	Lower	48.97%	
					Two-tailed	0.868	Upper	64.02%	
							Bayesian point	56.58%	
			TP4	0.800	One-tailed	0.407	Lower	33.41%	
					Two-tailed	0.815	Upper	48.34%	
							Bayesian point	40.74%	
		**Twin B**	TP1	1.200	One-tailed	0.434	Lower	48.95%	
					Two-tailed	0.868	Upper	63.99%	
							Bayesian point	56.58%	
			TP2	0.200	One-tailed	0.203	Lower	14.51%	
					Two-tailed	0.405	Upper	26.82%	
							Bayesian point	20.26%	
			TP3	0.400	One-tailed	0.263	Lower	19.89%	
					Two-tailed	0.527	Upper	33.42%	
							Bayesian point	26.34%	
			TP4	0.000	One-tailed	0.151	Lower	10.10%	
					Two-tailed	0.302	Upper	21.11%	
							Bayesian point	15.12%	

Supplementary Information S3: Longitudinal Assessment of Heart Rate Variability Parameters for Twin A (pre and post buspirone initiation)

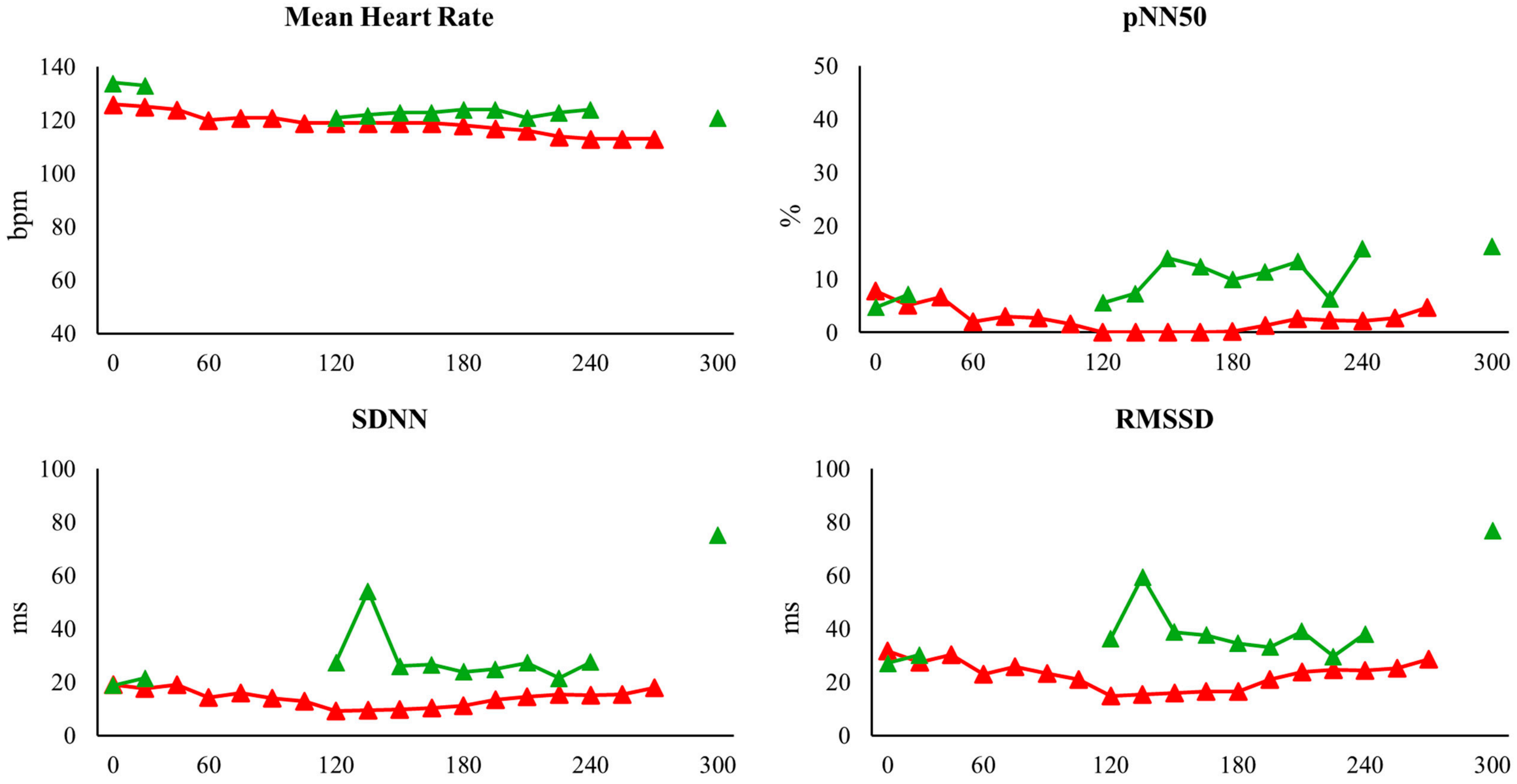

Legend:

▲ Before the initiation of buspirone ▲ After the initiation of buspirone

Abbreviations: bpm (beats per minute); ms (milliseconds)

Supplementary Information S4: Genotype Results

*MECP2**

Twin A

Gene	Zygosity	Inheritance	HGVS description	Location: GRCh37 (hg19)	Classification
MECP2	Heterozygous	*De novo*	NM_004992.3: c.1160_*5215del p(Pro387_Ser486delinsGln)	ChrX: g.153290603_153296119del	Likely pathogenic - consistent with a diagnosis of RTT

Twin B

Gene	Zygosity	Inheritance	HGVS description	Location: GRCh37 (hg19)	Classification
MECP2	Heterozygous	*De novo*	NM_004992.3: c.1160_*5215del p(Pro387_Ser486delinsGln)	ChrX: g.153290603_153296119del	Likely pathogenic - consistent with a diagnosis of RTT

*Information obtained from genomic laboratory report

*BDNF**

Twin A

Gene	Genotype	Phenotype	Clinical consequences
BDNF	434C>T C/T	Heterozygous for rs6265 T allele	Associated with reduced activity-dependent secretion of BDNF from neurons and impaired BDNF signaling.

Twin B

Gene	Genotype	Phenotype	Clinical consequences
BDNF	434C>T C/C	Homozygous for rs6265 C allele	Associated with normal activity-dependent secretion of BDNF from neurons and normal BDNF signaling

*Information obtained from pharmacogenetic report

Abbreviations: BDNF (brain-derived neurotrophic factor); ChrX (chromosome X); GRCh37 (Genome Reference Consortium Human Build 37); HG19 (Human Genome version 19); HGVS (Human Genome Variation Society); *MECP2* (methyl CpG binding protein 2); RTT (Rett Syndrome)
